# Synthesis, structure and Hirshfeld surface analysis of 2-oxo-2*H*-chromen-4-yl penta­noate

**DOI:** 10.1107/S205698902400584X

**Published:** 2024-06-21

**Authors:** Valentin Bationo, Konan René Kambo, Charles Bavouma Sombié, Rasmané Semdé, Pierre Francotte, Abdoulaye Djandé

**Affiliations:** a Department of Chemistry, Doctoral School of Sciences and Technology, University Joseph KI-ZERBO, Laboratory of Molecular Chemistry and Materials, Research Team: Organic Chemistry and Phytochemistry, 03 BP 7021 Ouagadougou 03, Burkina Faso; b Laboratory of Environmental Science and Technology University Jean Lorougnon GUEDE of Daloa BP 150 Daloa Côte d’Ivoire; c Doctoral School of Sciences and Health, University Joseph KI-ZERBO, Laboratory of Drug Development Center of Training, Research and Expertise in Pharmaceutical Sciences (CFOREM), 03 BP 7021 Ouagadougou 03, Burkina Faso; d Center for Interdisciplinary Research on Medicinal Chemistry, University of Liège, Avenue Hippocrate 15 (B36), B-4000, Liège, Belgium; University of Hyogo, Japan

**Keywords:** crystal structure, coumarin structure, Hirshfeld surface analysis, inter­actions, hydrogen bonds

## Abstract

In the title compound, C_14_H_14_O_4_, the dihedral angle between the coumarin ring system (r.m.s deviation = 0.016 Å) and the penta­noate ring is 36.26 (8)°. A short intra­molecular C—H⋯O contact of 2.40 Å is observed. Hirshfeld surface analysis reveals that 46.1% of the inter­molecular inter­actions are from H⋯H contacts, 28.6% are from H⋯O/O⋯H contacts and 14.7% are from H⋯C/C⋯H.

## Abstract 2

In the title compound, the dihedral angle between the coumarin ring system and the penta­noate ring is 36.26 (8)°. A short intra­molecular C—H⋯O contact is observed.

## 1. Chemical context

Coumarins are naturally occurring mol­ecules with a versatile range of activities. Their structural and physicochemical characteristics make them a privileged scaffold in medicinal chemistry and chemical biology (Carneiro *et al.*, 2021 ▸). Historically, coumarins have been applied for the treatment of a variety of diseases due to their anti­coagulant, anti-inflammatory, anti­viral, anti­microbial, anti­cancer, anti­oxidant (Todorov *et al.*, 2023 ▸) and anti-glaucoma (Ziki *et al.*, 2023 ▸) activities. Their wide range of biological activities and the use of coumarin-containing drugs clinically have contributed to the growing inter­est in this class of heterocycles (Khandy *et al.*, 2024 ▸). Given their importance, coumarin derivatives continue to be our field of research (Kambo *et al.*, 2017 ▸; Hollauer *et al.*, 2023 ▸). We report herein the synthesis, crystal structure, and Hirshfeld surface analysis of the title coumarin derivative.
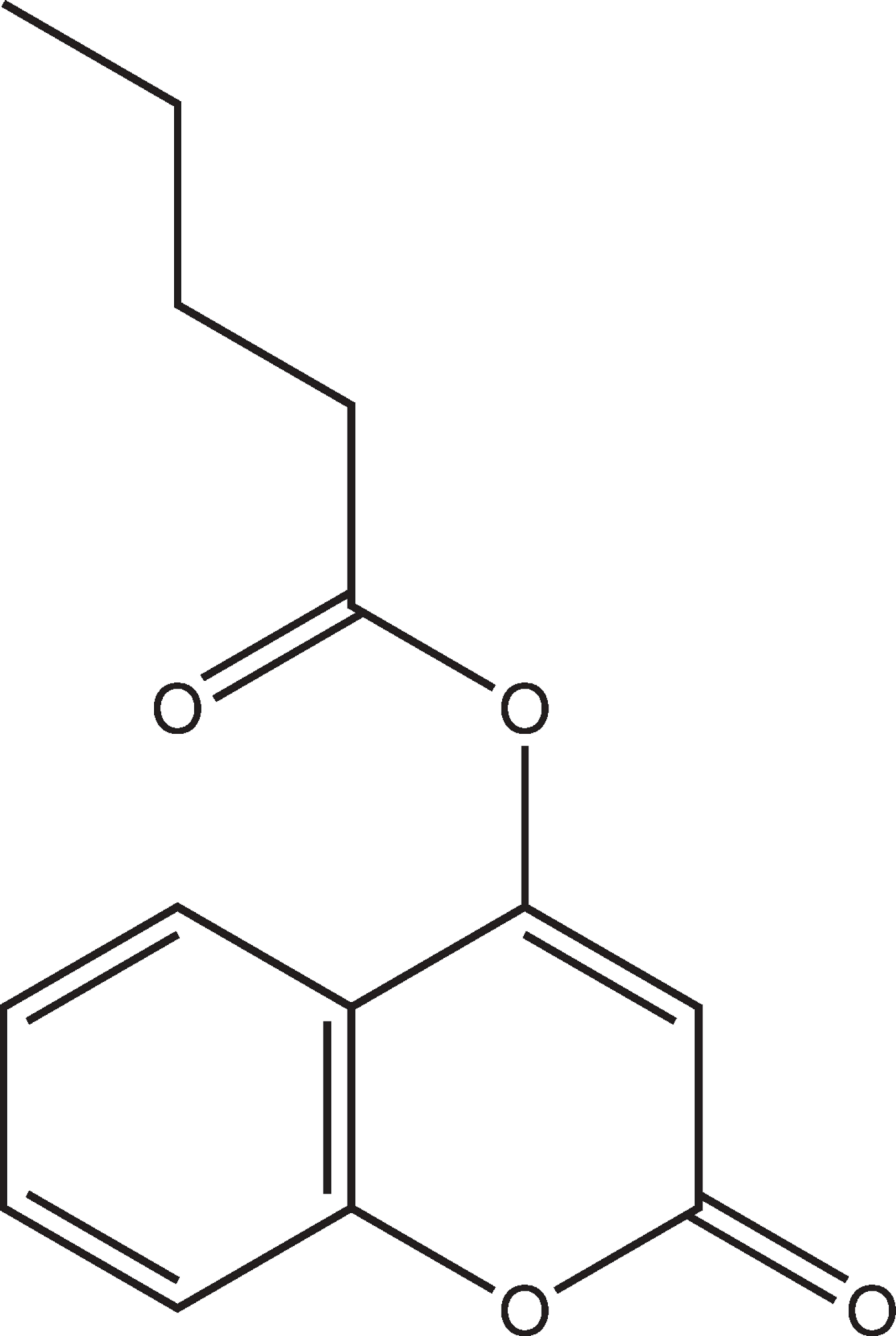


## 2. Structural commentary

**Figure 1:**
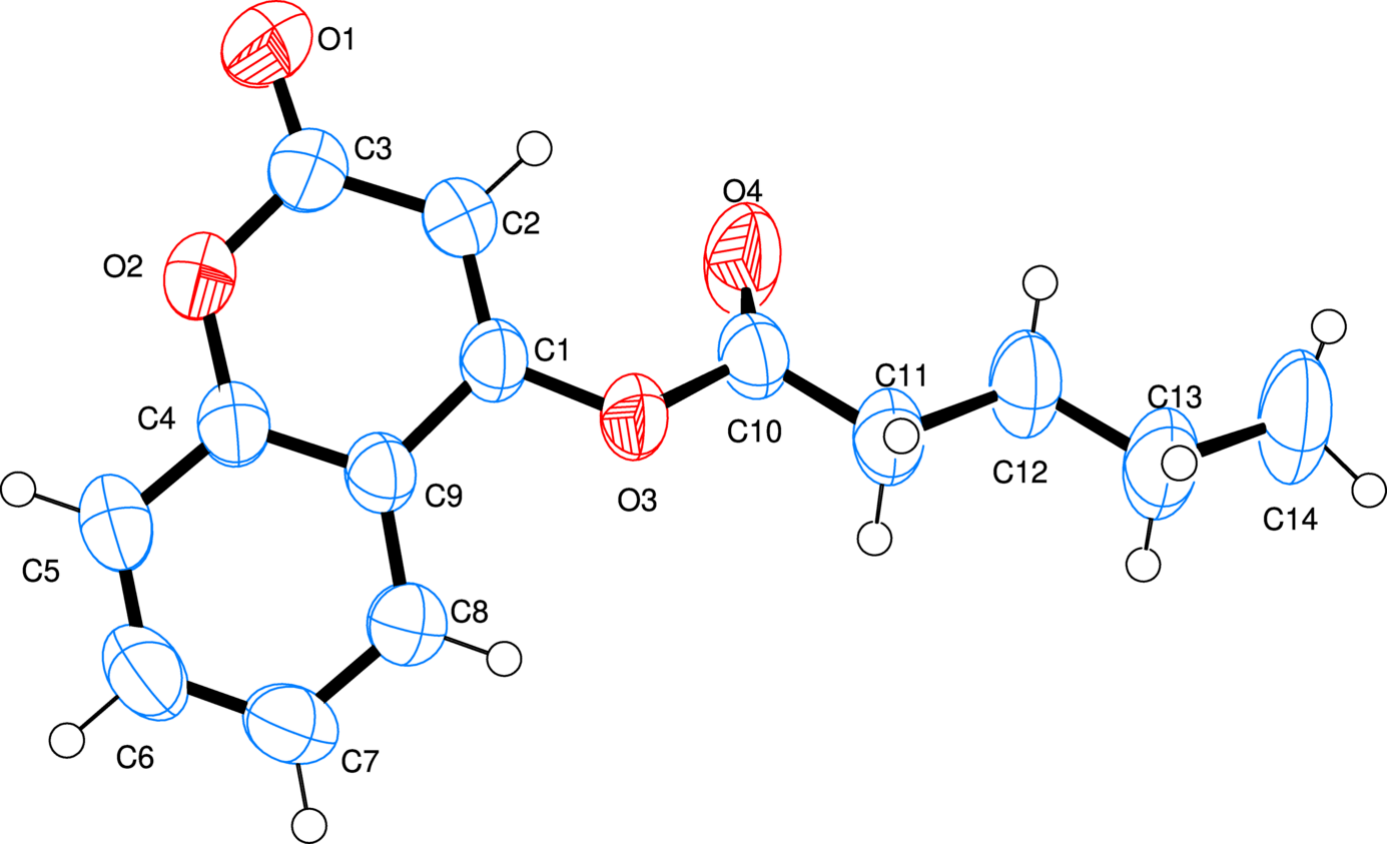
Mol­ecular structure of the compound showing the atomic numbering system. Displacement ellipsoids are drawn at the 50% probability level.

The mol­ecular structure of the title coumarin derivative is illustrated in Fig. 1 ▸. An *S*(6) ring motif arises from an intra­molecular C2—H2⋯O4 hydrogen bond (Table 1 ▸). As expected, the coumarin ring system is almost planar, with a maximum deviation from the plane of 0.016 (3) Å for atom C7. An inspection of the bond lengths shows that there is a slight asymmetry of the electronic distribution around the pyrone ring: the C1—C2 [1.336 (3) Å] and C2—C3 [1.437 (3) Å] bond lengths are shorter and longer, respectively, than those excepted for a C_ar_—C_ar_ bond. This suggests that the electron density is preferentially located in the C1—C2 bond of the pyrone ring, as seen in other coumarin derivatives (Gomes *et al.*, 2016 ▸; Ouédraogo *et al.*, 2018 ▸).

## 3. Supra­molecular features and Hirshfeld surface analysis

**Figure 2:**
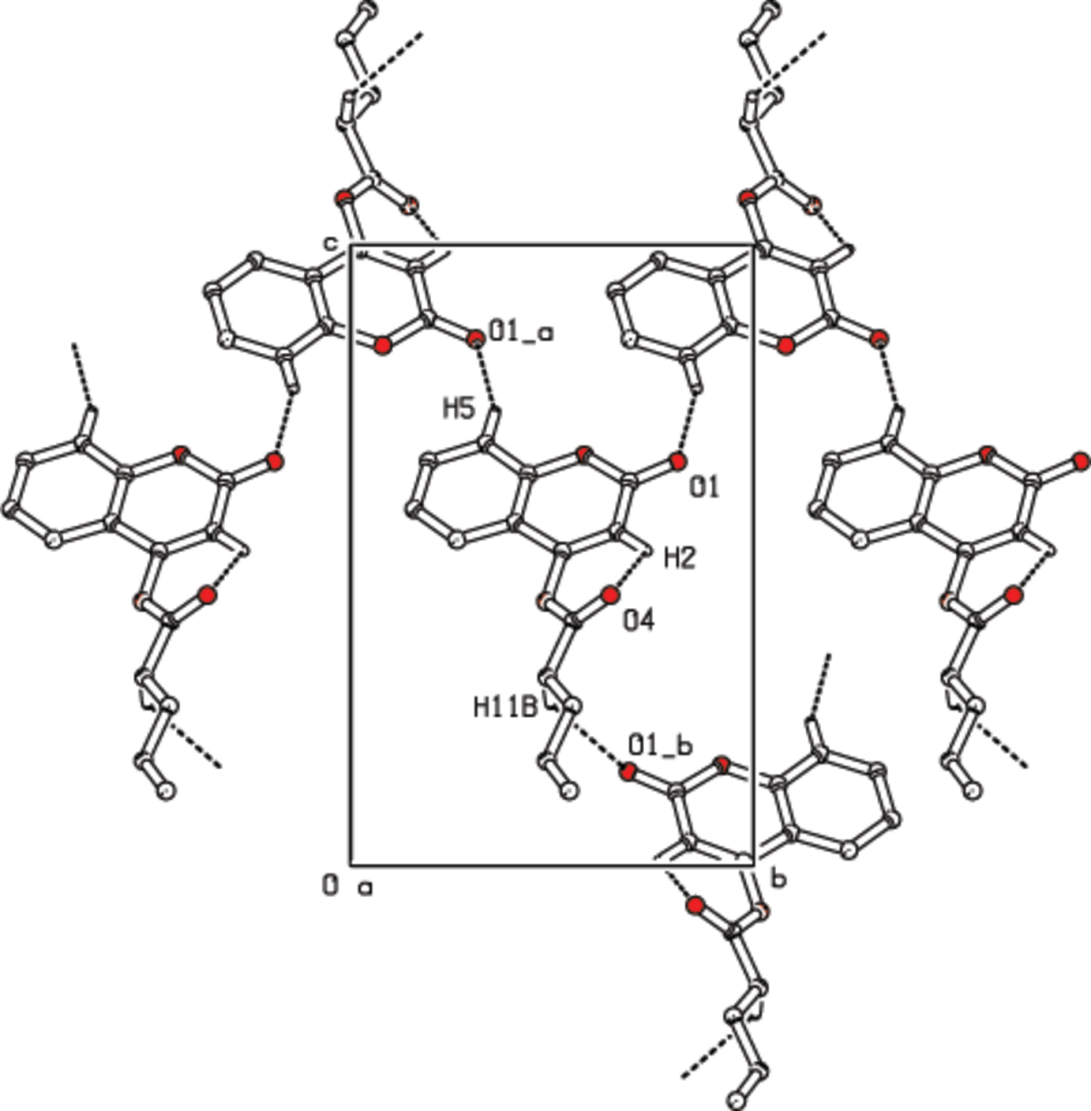
Part of crystalline packing of the title compound showing a parallel chain in the [001] direction. Dashed lines indicate hydrogen bonds. H atoms not involved in hydrogen-bonding inter­actions have been omitted for clarity.

**Figure 3:**
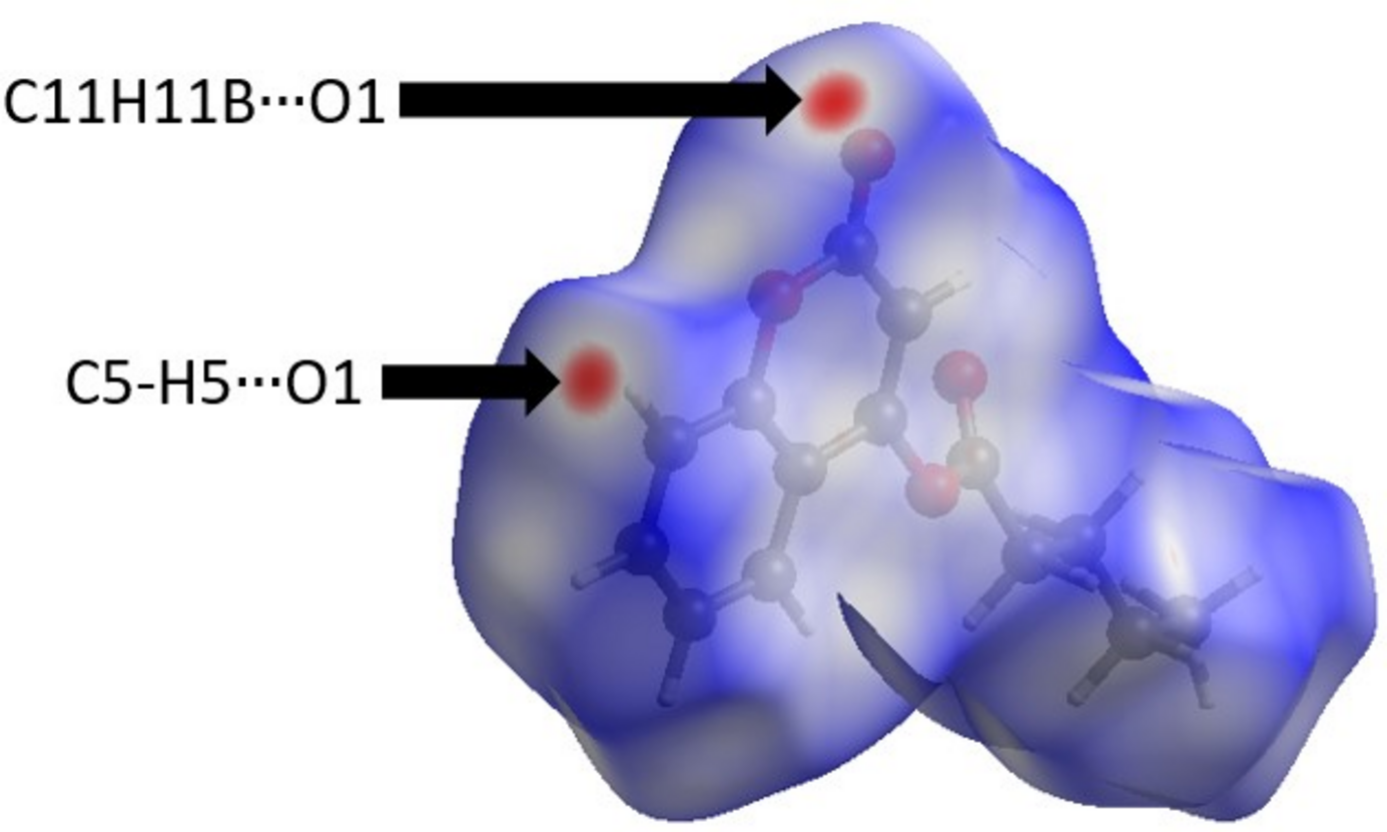
The Hirshfeld surface mapped over *d*_norm_ for visualizing the inter­molecular contacts of the title compound.

**Figure 4:**
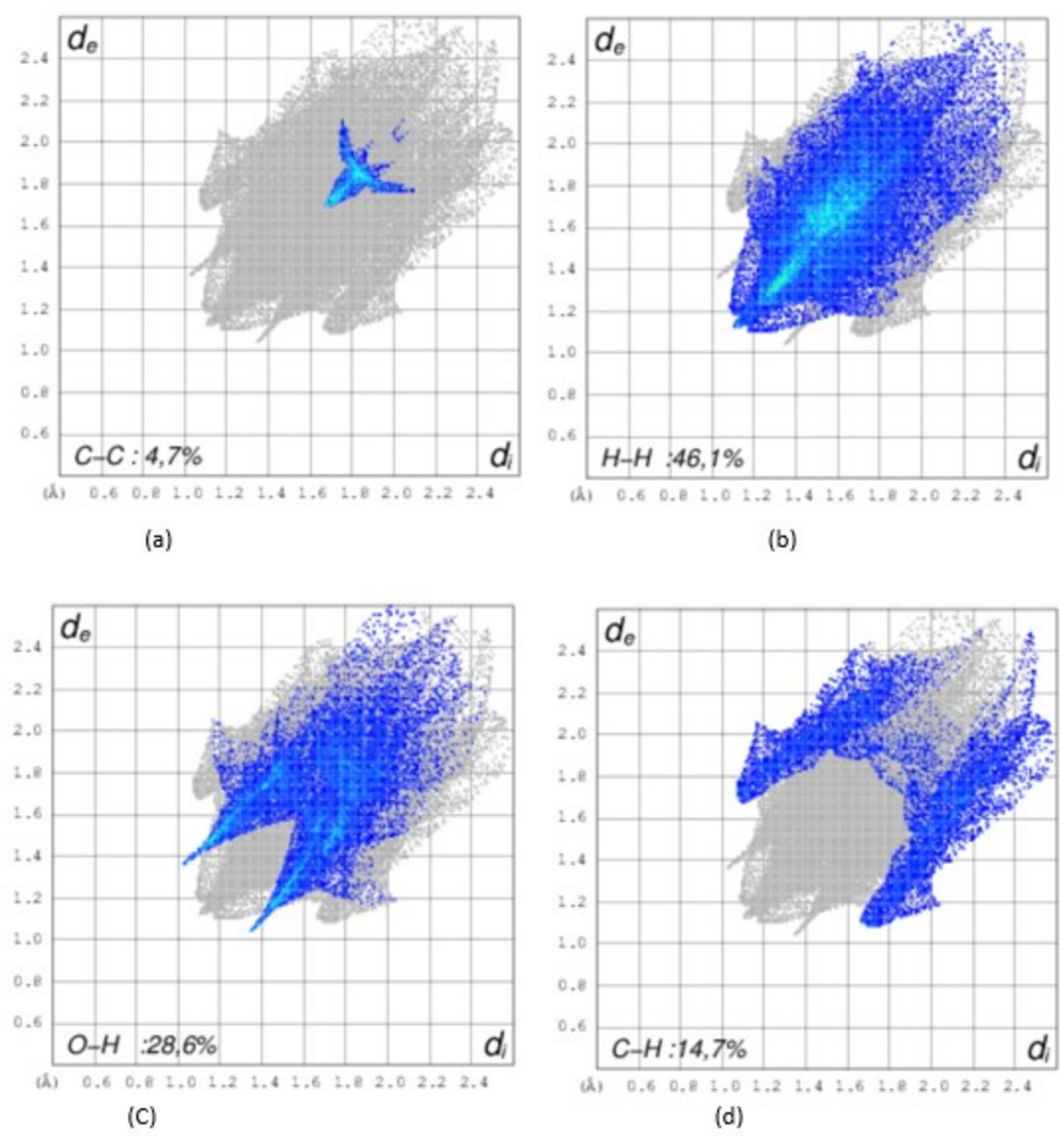
Fingerprint plots for the title compound showing (*a*) C⋯C, (*b*) H⋯H, (*c*) O⋯H/H⋯O and (*d*) C⋯H/H⋯C inter­actions. The outline of the full fingerprint is shown in grey. *d*_i_ is the closest inter­nal distance from a given point on the Hirshfeld surface and *d*_e_ is the closest external contact.

In the crystal, C5—H5⋯O1 hydrogen bonds link mol­ecules into infinite chains along the [001] direction (Table 1 ▸, Fig. 2 ▸) and the C11—H11*B*⋯O1 inter­actions contribute to the crystal cohesion. The inter­molecular inter­actions were qu­an­ti­fied using Hirshfeld surface analysis. This approach is a graphical tool for visualization and understanding of inter­molecular inter­actions. The Hirshfeld surface analysis was performed, and the two-dimensional (2D) fingerprint plots were generated with *CrystalExplorer 17* (Spackman *et al.*, 2021 ▸). Fig. 3 ▸ shows the Hirshfeld surface plotted over *d*_norm_ (normalized contact distance) and Fig. 4 ▸ the 2D fingerprint plots..

## 4. Database survey

A search of the Cambridge structural Database (CSD; Groom *et al.*, 2016 ▸; updated to April 2024) found seven coumarins structures with substituents at the 4-positions (XUFGOW, Kavitha *et al.*, 2015 ▸; NUZJOJ, Vinduvahini *et al.*, 2016 ▸; UDOGIF01, Anitha *et al.*, 2016 ▸, HUYVEE, Anitha *et al.*, 2015 ▸; NAGWAW, Ravi *et al.*, 2016 ▸; DIWPAE, Hollauer *et al.*, 2023 ▸). All seven have structural parameters very similar to this one, including essentially planar chromene portions.

## 5. Synthesis and crystallization

To a solution of valeroyl chloride (6.17 mmol, ∼0.8 ml) in dried diethyl ether (16 ml) was added dried pyridine (2.31 ml; 4.7 molar equivalents) and 4-hy­droxy­coumarin (6.17 mmol, 1 g) in small portions over 30 min, with vigorous stirring. The reaction mixture was left stirring at room temperature for 3 h.

The mixture was then poured in a separating funnel containing 40 ml of chloro­form and washed with diluted hydro­chloric acid solution until the pH was 2–3. The organic layer was extracted, washed with water to neutrality, dried over MgSO_4_ and the solvent removed. The crude product was filtered off with suction, washed with *n*-hexane and recrystallized from acetone. Dirty white crystals of the title compound were obtained in a good yield (78%), m.p. 408–409 K.

## 6. Refinement details

Crystal data, data collection and structure refinement details are summarized in Table 2 ▸. Hydrogen atoms were located in a difference-Fourier map, but were positioned with idealized geometry and refined isotropically using a riding model (HFIX command), *U*_iso_(H) = 1.5*U*_eq_(C-meth­yl) and 1.2*U*_eq_(C) for all other H atoms.

## Supplementary Material

Crystal structure: contains datablock(s) I. DOI: 10.1107/S205698902400584X/ox2005sup1.cif

Structure factors: contains datablock(s) I. DOI: 10.1107/S205698902400584X/ox2005Isup3.hkl

Supporting information file. DOI: 10.1107/S205698902400584X/ox2005Isup3.cml

CCDC reference: 2363131

Additional supporting information:  crystallographic information; 3D view; checkCIF report

## Figures and Tables

**Table 1:** Hydrogen-bond geometry (Å, °)

*D*—H⋯*A*	*D*—H	H⋯*A*	*D*⋯*A*	*D*—H⋯*A*
C2—H2⋯O4	0.93	2.40	2.855 (3)	110
C5—H5⋯O1^i^	0.93	2.53	3.387 (3)	153
C11—H11*B*⋯O1^ii^	0.97	2.65	3.446 (3)	139

Symmetry codes: (i) 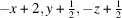; (ii) 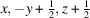.

**Table 2:** Experimental details

Crystal data	
Chemical formula	C_14_H_14_O_4_
*M*_r_	246.25
Crystal system, space group	Monoclinic, *P*2_1_/*c*
Temperature (K)	295
*a*, *b*, *c* (Å)	9.57455 (13), 9.29660 (17), 14.5761 (2)
β (°)	100.9517 (14)
*V* (Å^3^)	1273.80 (3)
*Z*	4
Radiation type	Cu *K*α
μ (mm^−1^)	0.78
Crystal size (mm)	0.26 × 0.22 × 0.18

Data collection	
Diffractometer	Rigaku Oxford Diffraction SuperNova, Dual, Atlas 2
Absorption correction	Multi-scan (*CrysAlis PRO*; Rigaku OD, 2023 ▸)
*T*_min_, *T*_max_	0.869, 1.000
No. of measured, independent and observed [*I* > 2σ(*I*)] reflections	13663, 2492, 2107
*R*_int_	0.024
(sin θ/λ)_max_ (Å^−1^)	0.618

Refinement	
*R*[*F*^2^ > 2σ(*F*^2^)], *wR*(*F*^2^), *S*	0.056, 0.175, 1.08
No. of reflections	2492
No. of parameters	164
H-atom treatment	H-atom parameters constrained
Δρ_max_, Δρ_min_ (e Å^−3^)	0.30, −0.27

Computer programs: *CrysAlis PRO* (Rigaku OD, 2023 ▸), *SHELXT* (Sheldrick, 2015*a* ▸), *SHELXL2013* (Sheldrick, 2015*b* ▸), *PLATON* (Spek, 2020 ▸), *ORTEP*-III Burnett & Johnson, 1996 ▸), *WinGX* (Farrugia, 2012 ▸) and *publCIF* (Westrip, 2010 ▸).

## supplementary crystallographic information

### 2-Oxo-2H-chromen-4-yl pentanoate Crystal data

**Table:**

C_14_H_14_O_4_	*F*(000) = 520
*M**_r_* = 246.25	*D*_x_ = 1.284 Mg m^−^^3^
Monoclinic, *P*2_1_/*c*	Cu *K*α radiation, λ = 1.54184 Å
*a* = 9.57455 (13) Å	Cell parameters from 5988 reflections
*b* = 9.29660 (17) Å	θ = 6.2–72.3°
*c* = 14.5761 (2) Å	µ = 0.78 mm^−^^1^
β = 100.9517 (14)°	*T* = 295 K
*V* = 1273.80 (3) Å^3^	Prism, colourless
*Z* = 4	0.26 × 0.22 × 0.18 mm

### 2-Oxo-2H-chromen-4-yl pentanoate Data collection

**Table:**

Rigaku Oxford Diffraction SuperNova, Dual, Atlas 2 diffractometer	2492 independent reflections
Radiation source: micro-focus sealed X-ray tube	2107 reflections with *I* > 2σ(*I*)
Mirror monochromator	*R*_int_ = 0.024
Detector resolution: 5.3045 pixels mm^-1^	θ_max_ = 72.4°, θ_min_ = 4.7°
ω scan	*h* = −11→11
Absorption correction: multi-scan (CrysAlisPro; Rigaku OD, 2023)	*k* = −11→9
*T*_min_ = 0.869, *T*_max_ = 1.000	*l* = −18→17
13663 measured reflections	

### 2-Oxo-2H-chromen-4-yl pentanoate Refinement

**Table:**

Refinement on *F*^2^	0 restraints
Least-squares matrix: full	Hydrogen site location: inferred from neighbouring sites
*R*[*F*^2^ > 2σ(*F*^2^)] = 0.056	H-atom parameters constrained
*wR*(*F*^2^) = 0.175	*w* = 1/[σ^2^(*F*_o_^2^) + (0.0899*P*)^2^ + 0.3573*P*] where *P* = (*F*_o_^2^ + 2*F*_c_^2^)/3
*S* = 1.08	(Δ/σ)_max_ < 0.001
2492 reflections	Δρ_max_ = 0.30 e Å^−^^3^
164 parameters	Δρ_min_ = −0.27 e Å^−^^3^

### 2-Oxo-2H-chromen-4-yl pentanoate Special details

**Table:**

Experimental. CrysAlisPro 1.171.42.102a (Rigaku Oxford Diffraction, 2023) Empirical absorption correction using spherical harmonics, implemented in SCALE3 ABSPACK scaling algorithm.
Geometry. All esds (except the esd in the dihedral angle between two l.s. planes) are estimated using the full covariance matrix. The cell esds are taken into account individually in the estimation of esds in distances, angles and torsion angles; correlations between esds in cell parameters are only used when they are defined by crystal symmetry. An approximate (isotropic) treatment of cell esds is used for estimating esds involving l.s. planes.

### 2-Oxo-2H-chromen-4-yl pentanoate Fractional atomic coordinates and isotropic or equivalent isotropic displacement parameters (Å^2^)

**Table:**

	*x*	*y*	*z*	*U*_iso_*/*U*_eq_	
O1	0.8531 (2)	0.18982 (18)	0.34863 (13)	0.0878 (6)	
O2	0.89842 (16)	0.42028 (16)	0.33853 (10)	0.0657 (4)	
O3	0.73548 (14)	0.51487 (15)	0.57413 (9)	0.0611 (4)	
O4	0.5562 (2)	0.3555 (2)	0.56376 (12)	0.0961 (7)	
C1	0.78028 (18)	0.4741 (2)	0.49394 (12)	0.0521 (4)	
C2	0.7829 (2)	0.3396 (2)	0.46205 (14)	0.0601 (5)	
H2	0.7447	0.2654	0.4923	0.072*	
C3	0.8442 (2)	0.3080 (2)	0.38166 (15)	0.0637 (5)	
C4	0.89359 (19)	0.5596 (2)	0.36974 (13)	0.0552 (5)	
C5	0.9492 (2)	0.6653 (3)	0.32029 (15)	0.0686 (6)	
H5	0.9891	0.6417	0.2689	0.082*	
C6	0.9443 (3)	0.8055 (3)	0.34855 (18)	0.0770 (7)	
H6	0.9807	0.8776	0.3155	0.092*	
C7	0.8862 (3)	0.8417 (2)	0.42546 (19)	0.0769 (6)	
H7	0.8837	0.9373	0.4439	0.092*	
C8	0.8321 (2)	0.7356 (2)	0.47459 (15)	0.0640 (5)	
H8	0.7930	0.7600	0.5263	0.077*	
C9	0.83527 (18)	0.5928 (2)	0.44771 (13)	0.0514 (4)	
C10	0.6263 (2)	0.4471 (2)	0.60627 (13)	0.0588 (5)	
C11	0.6104 (3)	0.5117 (3)	0.69639 (16)	0.0763 (7)	
H11A	0.5905	0.6134	0.6862	0.092*	
H11B	0.7010	0.5039	0.7392	0.092*	
C12	0.4994 (3)	0.4499 (3)	0.74266 (16)	0.0776 (7)	
H12A	0.4091	0.4569	0.6995	0.093*	
H12B	0.5199	0.3484	0.7531	0.093*	
C13	0.4818 (4)	0.5132 (4)	0.8311 (2)	0.1043 (11)	
H13A	0.4636	0.6150	0.8203	0.125*	
H13B	0.5723	0.5050	0.8739	0.125*	
C14	0.3720 (4)	0.4574 (4)	0.8790 (2)	0.1154 (12)	
H14A	0.2833	0.4508	0.8355	0.173*	
H14B	0.3614	0.5211	0.9291	0.173*	
H14C	0.3992	0.3637	0.9038	0.173*	

### 2-Oxo-2H-chromen-4-yl pentanoate Atomic displacement parameters (Å^2^)

**Table:**

	*U*^11^	*U*^22^	*U*^33^	*U*^12^	*U*^13^	*U*^23^
O1	0.1124 (13)	0.0674 (10)	0.0945 (12)	−0.0079 (9)	0.0470 (10)	−0.0231 (9)
O2	0.0772 (9)	0.0669 (9)	0.0606 (8)	−0.0033 (7)	0.0327 (7)	−0.0066 (6)
O3	0.0666 (8)	0.0655 (9)	0.0593 (8)	−0.0121 (6)	0.0321 (6)	−0.0060 (6)
O4	0.0994 (12)	0.1166 (15)	0.0855 (11)	−0.0483 (11)	0.0512 (10)	−0.0319 (10)
C1	0.0498 (8)	0.0594 (11)	0.0511 (9)	−0.0023 (7)	0.0197 (7)	0.0002 (8)
C2	0.0676 (11)	0.0539 (11)	0.0645 (11)	−0.0058 (9)	0.0274 (9)	0.0005 (9)
C3	0.0685 (11)	0.0610 (12)	0.0661 (12)	−0.0034 (9)	0.0237 (9)	−0.0052 (9)
C4	0.0546 (9)	0.0614 (11)	0.0523 (9)	0.0002 (8)	0.0174 (7)	0.0041 (8)
C5	0.0726 (12)	0.0783 (14)	0.0605 (11)	−0.0048 (10)	0.0269 (10)	0.0116 (10)
C6	0.0829 (15)	0.0721 (15)	0.0812 (15)	−0.0046 (11)	0.0286 (12)	0.0243 (12)
C7	0.0876 (15)	0.0513 (12)	0.0967 (17)	−0.0010 (10)	0.0302 (13)	0.0088 (11)
C8	0.0666 (11)	0.0582 (12)	0.0728 (12)	0.0017 (9)	0.0276 (10)	0.0010 (10)
C9	0.0479 (8)	0.0549 (11)	0.0545 (9)	0.0003 (7)	0.0175 (7)	0.0036 (8)
C10	0.0585 (10)	0.0657 (12)	0.0568 (10)	−0.0064 (9)	0.0224 (8)	0.0035 (9)
C11	0.0903 (15)	0.0834 (16)	0.0652 (12)	−0.0196 (12)	0.0402 (11)	−0.0079 (11)
C12	0.0740 (13)	0.1044 (18)	0.0614 (12)	−0.0191 (13)	0.0304 (10)	−0.0062 (12)
C13	0.131 (2)	0.116 (2)	0.0846 (17)	−0.0419 (19)	0.0664 (17)	−0.0261 (16)
C14	0.117 (2)	0.167 (3)	0.0780 (17)	−0.043 (2)	0.0579 (17)	−0.0214 (19)

### 2-Oxo-2H-chromen-4-yl pentanoate Geometric parameters (Å, º)

**Table:**

O1—C3	1.209 (3)	C7—H7	0.9300
O2—C3	1.371 (2)	C8—C9	1.387 (3)
O2—C4	1.377 (2)	C8—H8	0.9300
O3—C1	1.373 (2)	C10—C11	1.478 (3)
O3—C10	1.377 (2)	C11—C12	1.479 (3)
O4—C10	1.184 (3)	C11—H11A	0.9700
C1—C2	1.336 (3)	C11—H11B	0.9700
C1—C9	1.443 (2)	C12—C13	1.455 (3)
C2—C3	1.437 (3)	C12—H12A	0.9700
C2—H2	0.9300	C12—H12B	0.9700
C4—C5	1.384 (3)	C13—C14	1.464 (3)
C4—C9	1.393 (3)	C13—H13A	0.9700
C5—C6	1.370 (3)	C13—H13B	0.9700
C5—H5	0.9300	C14—H14A	0.9600
C6—C7	1.384 (4)	C14—H14B	0.9600
C6—H6	0.9300	C14—H14C	0.9600
C7—C8	1.376 (3)		
			
C3—O2—C4	121.71 (15)	C4—C9—C1	116.78 (17)
C1—O3—C10	122.95 (15)	O4—C10—O3	122.85 (18)
C2—C1—O3	125.75 (17)	O4—C10—C11	127.85 (18)
C2—C1—C9	121.34 (17)	O3—C10—C11	109.25 (17)
O3—C1—C9	112.80 (16)	C10—C11—C12	116.9 (2)
C1—C2—C3	120.85 (18)	C10—C11—H11A	108.1
C1—C2—H2	119.6	C12—C11—H11A	108.1
C3—C2—H2	119.6	C10—C11—H11B	108.1
O1—C3—O2	116.60 (19)	C12—C11—H11B	108.1
O1—C3—C2	125.5 (2)	H11A—C11—H11B	107.3
O2—C3—C2	117.86 (18)	C13—C12—C11	117.4 (2)
O2—C4—C5	117.11 (17)	C13—C12—H12A	107.9
O2—C4—C9	121.44 (17)	C11—C12—H12A	107.9
C5—C4—C9	121.45 (19)	C13—C12—H12B	107.9
C6—C5—C4	118.7 (2)	C11—C12—H12B	107.9
C6—C5—H5	120.6	H12A—C12—H12B	107.2
C4—C5—H5	120.6	C12—C13—C14	119.6 (3)
C5—C6—C7	121.1 (2)	C12—C13—H13A	107.4
C5—C6—H6	119.5	C14—C13—H13A	107.4
C7—C6—H6	119.5	C12—C13—H13B	107.4
C8—C7—C6	119.8 (2)	C14—C13—H13B	107.4
C8—C7—H7	120.1	H13A—C13—H13B	107.0
C6—C7—H7	120.1	C13—C14—H14A	109.5
C7—C8—C9	120.5 (2)	C13—C14—H14B	109.5
C7—C8—H8	119.7	H14A—C14—H14B	109.5
C9—C8—H8	119.7	C13—C14—H14C	109.5
C8—C9—C4	118.44 (17)	H14A—C14—H14C	109.5
C8—C9—C1	124.78 (17)	H14B—C14—H14C	109.5
			
C10—O3—C1—C2	−34.4 (3)	C7—C8—C9—C1	−179.4 (2)
C10—O3—C1—C9	149.38 (17)	O2—C4—C9—C8	−179.15 (17)
O3—C1—C2—C3	−174.29 (19)	C5—C4—C9—C8	0.9 (3)
C9—C1—C2—C3	1.6 (3)	O2—C4—C9—C1	0.0 (3)
C4—O2—C3—O1	179.69 (19)	C5—C4—C9—C1	179.97 (17)
C4—O2—C3—C2	−0.8 (3)	C2—C1—C9—C8	177.8 (2)
C1—C2—C3—O1	178.9 (2)	O3—C1—C9—C8	−5.9 (3)
C1—C2—C3—O2	−0.5 (3)	C2—C1—C9—C4	−1.3 (3)
C3—O2—C4—C5	−178.94 (18)	O3—C1—C9—C4	175.07 (16)
C3—O2—C4—C9	1.1 (3)	C1—O3—C10—O4	−5.5 (3)
O2—C4—C5—C6	179.07 (19)	C1—O3—C10—C11	176.86 (19)
C9—C4—C5—C6	−0.9 (3)	O4—C10—C11—C12	3.8 (4)
C4—C5—C6—C7	0.5 (4)	O3—C10—C11—C12	−178.7 (2)
C5—C6—C7—C8	−0.1 (4)	C10—C11—C12—C13	−179.5 (3)
C6—C7—C8—C9	0.0 (4)	C11—C12—C13—C14	179.1 (3)
C7—C8—C9—C4	−0.4 (3)		

### 2-Oxo-2H-chromen-4-yl pentanoate Hydrogen-bond geometry (Å, º)

**Table:**

*D*—H···*A*	*D*—H	H···*A*	*D*···*A*	*D*—H···*A*
C2—H2···O4	0.93	2.40	2.855 (3)	110
C5—H5···O1^i^	0.93	2.53	3.387 (3)	153
C11—H11*B*···O1^ii^	0.97	2.65	3.446 (3)	139

Symmetry codes: (i) −*x*+2, *y*+1/2, −*z*+1/2; (ii) *x*, −*y*+1/2, *z*+1/2.
